# Pulmonary decellularized extracellular matrix (dECM) modified polyethylene terephthalate three-dimensional cell carriers regulate the proliferation and paracrine activity of mesenchymal stem cells

**DOI:** 10.3389/fbioe.2023.1324424

**Published:** 2024-01-08

**Authors:** Jinze Li, Jiali Zhang, Hao Ye, Qixuan Wang, Yanran Ouyang, Yuxi Luo, Yihong Gong

**Affiliations:** ^1^ School of Biomedical Engineering, Shenzhen Campus of Sun Yat-Sen University, Shenzhen, China; ^2^ Guangdong Provincial Key Laboratory of Sensor Technology and Biomedical Instrument, Sun Yat-Sen University, Guangzhou, China

**Keywords:** decellularized materials, mesenchymal stem cells, extracellular matrix, growth factor expression, tissue engineering

## Abstract

**Introduction:** Mesenchymal stem cells (MSCs) possess a high degree of self-renewal capacity and *in vitro* multi-lineage differentiation potential. Decellularized materials have garnered considerable attention due to their elevated biocompatibility, reduced immunogenicity, excellent biodegradability, and the ability to partially mimic the in vivo microenvironment conducive to cell growth. To address the issue of mesenchymal stem cells losing their stem cell characteristics during two-dimensional (2D) cultivation, this study established three-dimensional cell carriers modified with lung decellularized extracellular matrix and assessed its impact on the life activities of mesenchymal stem cells.

**Methods:** This study employed PET as a substrate material, grafting with polydopamine (PDA), and constructing a decellularized extracellular matrix (dECM) coating on its surface, thus creating the PET/PDA/dECM three-dimensional (3D) composite carrier. Subsequently, material characterization of the cellular carriers was conducted, followed by co-culturing with human umbilical cord mesenchymal stem cells *in vitro*, aiming to investigate the material’s impact on the proliferation and paracrine activity of mesenchymal stem cells.

**Results and Discussion:** Material characterization demonstrated successful grafting of PDA and dECM materials, and it had complete hydrophilicity, high porosity, and excellent mechanical properties. The material was rich in various ECM proteins (collagen I, collagen IV , laminin, fibronectin, elastin), indicating good biocompatibility. In long-term *in vitro* cultivation (14 days) experiments, the PET/PDA/dECM three-dimensional composite carrier significantly enhanced adhesion and proliferation of human umbilical cord-derived mesenchymal stem cells (HUCMSCs), with a proliferation rate 1.9 times higher than that of cells cultured on tissue culture polystyrene (TCPS) at day 14. Furthermore, it effectively maintained the stem cell characteristics, expressing specific antigens for HUCMSCs. Through qPCR, Western blot, and ELISA experiments, the composite carrier markedly promoted the expression and secretion of key cell factors in HUCMSCs. These results demonstrate that the PET/PDA/dECM composite carrier holds great potential for scaling up MSCs’ long-term *in vitro* cultivation and the production of paracrine factors.

## 1 Introduction

Mesenchymal stem cells (MSCs) are a type of multipotent stem cells originating from the mesoderm (Pittenger et al., 2019), possessing a high degree of self-renewal and the ability to differentiate into various cell types *in vitro*. MSCs can interact with immune cells and others by paracrine signaling, chemokines, and growth factors, exhibiting favorable functions such as anti-inflammatory effects, tissue repair, and cellular homeostatic regeneration (Song et al., 2020). These exceptional characteristics make them ideal seed cells in the field of tissue engineering. MSCs derived from umbilical cords present several advantages, including simple acquisition, higher cell purity, lower immunogenicity, reduced tumorigenicity, and absence of ethical concerns (Qiao et al., 2020), rendering them highly valuable for clinical applications. Traditional two-dimensional (2D) cultivation methods often lead to MSCs losing their stem cell characteristics, as prolonged culture time can result in reduced proliferative and differentiation capabilities. In contrast, three-dimensional (3D) cultivation expands the cell culture surface, providing more tissue-like structures and growth space. It effectively mimics the *in vivo* cellular microenvironment, facilitating cell-cell and cell-extracellular matrix interactions, thus promoting MSCs’ *in vitro* expansion, multi-lineage differentiation potential, and expression of bioactive factors (Collins et al., 2010; Qiu et al., 2015; Yin et al., 2020; Huang et al., 2022; Shi et al., 2022). This approach reduces the disparities between *in vitro* culture and real clinical conditions, allowing for more accurate assessment of stem cell characteristics and guidance of their *in vitro* differentiation and paracrine functions.

Decellularization technology is a crucial method for producing decellularized extracellular matrix (dECM) materials sourced from cells or tissues. It involves the physical, chemical, or biological removal of cellular components from tissues or organs, resulting in the extraction of the extracellular matrix and its three-dimensional structure. The dECM materials prepared using this method exhibit excellent biomechanical properties, biocompatibility, and low immunogenicity, making them biocompatible biomaterials (Morris et al., 2017). They significantly reduce the presence of exogenous antigens, thereby lowering the occurrence of rejection and inflammatory reactions. Simultaneously, the basic structure and bioactivity of the extracellular matrix are preserved, providing cells with an environment similar to that of the *in vivo* setting. This enables the expression of the extracellular matrix’s biological functions, effectively promoting cellular activities such as proliferation, migration, and differentiation (Liu et al., 2016; Tian et al., 2021). In current research, notable progress has been achieved in the utilization of decellularized extracellular matrix (dECM) for coating biomaterials (Zhang et al., 2022). Notably, hyaluronic acid-chondroitin sulfate hydrogels containing decellularized cartilage extracellular matrix particles exhibit excellent mechanical properties and hold promising prospects in the field of cartilage repair (Guo et al., 2023). Furthermore, the composite material of Poly 1,8 octanediol citrate (POC) and aortic dECM demonstrates remarkable potential in reducing thrombosis and promoting vascular re-endothelialization (Jiang et al., 2015). Additionally, various dECM scaffolds are employed in tissue engineering applications, encompassing skin, bone, nerve, and others (Zhang et al., 2022). It is worth noting that dECM from different sources may contain varying types and levels of bioactive molecules, necessitating careful consideration by researchers when selecting suitable sources. Moreover, during the material preparation process, the choice of decellularization methods may result in residual immunogenicity or structural damage to the extracellular matrix, and highlighting the need to select appropriate materials to enhance the limited mechanical strength of dECM. The lung tissue comprises a porous sac-like structure composed of numerous alveoli and various bronchioles, containing a considerable amount of proteins and polysaccharides. Research indicates that the complex structure and components of the lung tissue’s extracellular matrix can effectively enhance MSCs’ proliferation and the expression of their immune functions (Batlle and Massague, 2019). Hence, in this study, lung tissue was chosen as the source of decellularized extracellular matrix (dECM).

Polyethylene terephthalate (PET) is one of the most common polymers, known for its excellent mechanical properties, cost-effectiveness, and processability. Particularly, in its fibrous form, PET exhibits a high porosity, high specific surface area, and interconnected pore structure, making it widely used in tissue repair and biomedical fields (Gustafsson et al., 2012; Liang et al., 2013). However, the surface properties of PET are suboptimal, necessitating surface modifications to enhance its biocompatibility. Techniques such as plasma treatment, UV irradiation, grafting of biomacromolecules (collagen, albumin), and cell-recognition peptide sequences (RGD peptide) have been employed (Liu et al., 2013). Nevertheless, these modification methods still face challenges such as coating instability, non-specific cellular responses, and cytotoxicity.

Dopamine (DA) is a key biomolecular element responsible for the remarkable adhesion properties of mussels. It can undergo self-polymerization in weak alkaline solutions, resulting in the formation of a polydopamine (PDA) coating that mimics the chemical composition of the mussel plaque-matrix interface (Nicklisch and Waite, 2012). Numerous investigators have observed that the incorporation of polydopamine (PDA) onto scaffold materials can enhance the compatibility of the cell-biomaterial interface, thereby augmenting cell adhesion and viability on the scaffold (Li et al., 2009; Wei et al., 2023). Furthermore, the catechol moieties on the PDA coating can undergo Schiff base and/or Michael addition reactions, facilitating further reactions with amine and thiol groups in target molecules, thereby contributing to the secondary immobilization of other functional molecules.

The surface hydrophobicity and lack of active sites have constrained the further application of polyethylene terephthalate (PET). In addressing the deficiencies of existing modification methods, characterized by unstable coatings and a certain level of cytotoxicity, this study utilizes PET as a substrate, grafting PDA to ameliorate surface hydrophilicity. The PDA surface modification engenders a uniform and stable polymer coating, thereby enhancing the material’s resistance to contamination and biocompatibility. Subsequently, we introduced a decellularized lung tissue solution to form an extracellular matrix coating on the PET fiber scaffold, successfully creating a PET/PDA/dECM three-dimensional composite carrier. The morphological characteristics, hydrophilicity, elemental content, and structural composition of the composite carrier were analyzed. The composition of the extracellular matrix coating was investigated through immunofluorescence staining. Moreover, we conducted further research on cell adhesion, proliferation, stemness maintenance, and cytokine secretion of HUCMSCs on TCPS, PET/PDA and PET/PDA/dECM carriers. The results demonstrated that the prepared carrier material possessed favorable properties and was suitable for long-term *in vitro* cultivation of HUCMSCs.

## 2 Materials and methods

### 2.1 Preparation of decellularized lung tissue material

**FIGURE 1:**
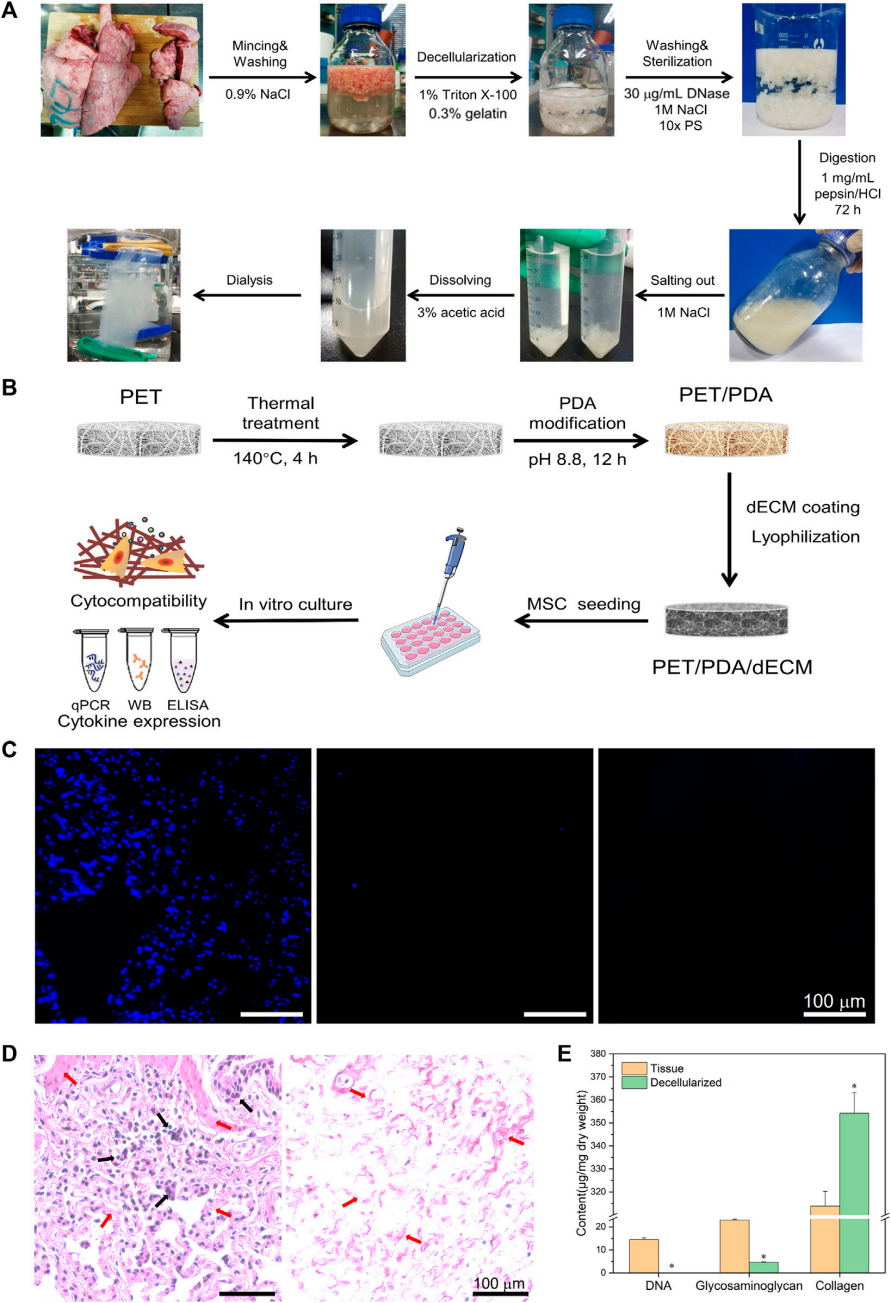
**(A)** Schematic diagram of porcine lung tissue decellularized material preparation process. **(B)** Schematic diagram of PET 3D carriers fabrication process. **(C)** Cell nucleus staining of porcine lung tissue before and after decellularization and enzymatic digestion. **(D)** Histological sections of tissue before and after decellularization stained with hematoxylin and eosin. Black arrows indicate the cellular nuclear structures, while red arrows point to the extracellular matrix structures. **(E)** Quantification of tissue DNA, glycosaminoglycans, and collagen before and after decellularization. Scale bars in **(C,D)** represent 100 μm (data = mean ± SD; *n* = 3; *p < 0.05).

After an exhaustive literature review, this study devised the following decellularization protocol (Zhang et al., 2023; Kerr et al., 2024). Fresh porcine distal trachea lungs tissue was cut into 3 × 3 cm pieces and washed with sterilized physiological saline (0.9% NaCl solution) to remove blood contamination. Immerse the tissue in a Penicillin-Streptomycin (Gibco, United States) solution at 20 times concentration. After cutting the tissue into small pieces, soak it in a 1% Triton X-100 (BioFroxx, Germany) solution and shake it on the shaker at room temperature for 24 h. Rinse the tissue with sterilized physiological saline. Next, place the tissue in a 0.3% gelatin (Sigma, United States) solution and shake it on the shaker for 1 h, followed by another rinse with sterilized physiological saline. Submerge the tissue in a 1 mol/L NaCl (Guangzhou Chemical Reagent Factory, China) solution and shake it on the shaker at room temperature for 1 h, then rinse with sterilized physiological saline. Immerse the tissue in a 30 μg/mg DNase (Sigma, United States) solution and shake it on a shaker for 4 h at room temperature, followed by a rinse with sterilized physiological saline. Soak the tissue in 10x PS and shake it on the shaker for 4 h, followed by a rinse with sterilized physiological saline. Digest the decellularized tissue using a 1 mg/mL pepsin (Sigma, United States)/0.01 mol/L hydrochloric acid (Guangzhou Chemical Reagent Factory, China) solution on the shaker for 72 h. After filtration, slowly drip the enzyme solution into a 1 M NaCl solution and let it stand for 30 min, then centrifuge at 2000 rpm for 10 min. Redissolve the precipitate in 3% acetic acid (Guangzhou Chemical Reagent Factory, China), dialyze it at 4°C for 2 days, and obtain the decellularized material. The procedure for preparing decellularized porcine lung tissue material is illustrated in Figure 1A.

### 2.2 Characterization of decellularized lung tissue material

#### 2.2.1 Hematoxylin and eosin (H&E) staining

The tissue was fixed with formaldehyde, dehydrated with ethanol, alcohol benzene, and xylene, embedded, and sectioned to a thickness of approximately 5 μm. After deparaffinization with xylene and ethanol to water, the sections were stained with hematoxylin for 5 min, followed by differentiation, rinsing, and bluing. Dehydration was performed using 85% and 100% ethanol for 6 min each, followed by staining with eosin for 6 min. Subsequently, the sections were dehydrated with ethanol and xylene to render transparent, and finally sealed with neutral resin.

#### 2.2.2 DAPI staining

DAPI staining solution was applied to the deparaffinized sections, and the staining was performed at room temperature in the dark for approximately 5 min. After staining, the sections were observed using a fluorescence microscope (IX71, Olympus, Japan).

#### 2.2.3 DNA quantification

The Hoechst 33,258 staining kit (APExBio, United States of America) was used to measure the DNA content in the tissue and tissue after decellularization. Logarithmic growth phase 3T3 cells were used as standard samples.

#### 2.2.4 Glycosaminoglycan (GAG) quantification

The DMMB assay kit (Sigma, United States) was used to detect the GAG content in the tissue before and after decellularization (Templeton, 1988). Chondroitin sulfate (Aladdin, United States) was used as the standard sample.

#### 2.2.5 Collagen quantification

The hydroxyproline assay kit (Shanghai Yuanye Bio-Technology Co., Ltd., China) was used to measure the collagen content in the tissue before and after decellularization (Ameer et al., 2002). Type I collagen was used as the standard sample.

### 2.3 Preparation of PET/PDA/dECM composite carrier

The PET sponge material (Yee, China) was cut (3 × 3 cm) and placed in a sealed container, then subjected to a 140°C oil bath treatment for 4 h. After the heat treatment, the PET was trimmed into discs with a diameter of approximately 15 mm and a thickness of 1–2 mm. These discs were then immersed in a dopamine ethanol solution for 1–2 h and subsequently drip-coated with Tris-HCl buffer (pH 8.8) under dark conditions overnight. After rinsing with deionized water, the modified carrier (PET/PDA) was obtained and dried in an oven. Next, the PET/PDA material was immersed in a solution comprising 40 mM 1-ethyl-3-(3-dimethylaminopropyl) carbodiimide (EDC, Sigma, United States), 20 mM N-hydroxysuccinimide (NHS, Sigma, United States), and 50 mM 2-[N-morpholino] ethanesulfonic acid (MES, Sigma, United States). An appropriate amount of dECM solution was added, and the mixture was incubated at 4°C for 24 h. Subsequently, the material was immersed in a 0.1 mol/L Na_2_HPO_4_ (Guangzhou Chemical Reagent Factory, China) solution for 1 h, followed by deionized water rinsing. After freezing at −20°C, the material was freeze-dried, resulting in the modified carrier (PET/PDA/dECM) with PDA and dECM modifications. The fabrication process of the PET 3D carriers are depicted in Figure 1B.

### 2.4 Characterization

#### 2.4.1 Surface morphology analysis of PET composite carrier

The samples were fixed onto the sample holder, sputter-coated with gold, and then placed in a vacuum chamber with a vacuum degree of 3 × 10^−3^ kPa. The acceleration voltage and current were set to 20 kV and 10 mA, respectively. The samples were observed under a scanning electron microscope (Quanta 400FEG, FEI, United States).

#### 2.4.2 Surface hydrophilicity testing of PET composite carrier

A droplet of 1–2 μL of water was added to the carrier, photographed, and then the contact angle between the solid and liquid phases was calculated.

#### 2.4.3 Determination of porosity in PET composite carrier

The porosity of the dried fiber carrier was determined using the following method (Wu et al., 2021):

$$Porosity\left(\%\right)=1-\;\frac{\rho_{0}}{\rho }\times 100\%$$ (1)

$\rho_{0}$ refers to the bulk density of the carrier, which is the density of the carrier in its natural state. ρ represents the density of the solid components of the carrier, i.e., the density under absolute compaction. Since the prepared carrier has a regular shape, its apparent volume can be calculated based on the base area multiplied by the height, while the absolute compact volume is measured using a pycnometer.

#### 2.4.4 Mechanical property testing of PET composite carrier

The tensile properties of the PET composite carrier were evaluated using a universal material testing machine (ZQ-990LB, Zhiqu, China), following the sample preparation standards outlined in GBT14344-2008. The specimen dimensions were as follows: an effective length of 75 mm, a parallel length of 32 mm, a width of 10 mm, a center width of 5 mm, and a thickness of approximately 1.5 mm in the form of a standard dumbbell shape. The material was immersed in a PBS solution before conducting the mechanical performance tests. The tensile rate was set at 20 mm/min, and each set of samples included three parallel specimens.

#### 2.4.5 Surface elemental analysis of PET composite carrier

X-ray photoelectron spectroscopy (XPS) was performed using an ESCALab250 instrument (Thermo Fisher, United States) to analyze the surface elements of the carriers treated with different modifications. The samples were cut into 3 × 3 mm pieces with a thickness of 1 mm. The analysis chamber maintained a working vacuum of approximately 2 × 10^−7^ Pa, and the X-ray source used an Al K source with an energy of 1486.6 eV, 15 kV, and 150 W. The scanning range was set from 0 to 1350 eV to measure the elements (C, O, N) and their content on the carrier.

#### 2.4.6 Chemical structure analysis of PET composite carrier

Different carriers were analyzed using Fourier-transform infrared (FTIR) spectrometer (Nicolet 6700, Thermo Fisher, United States). The wavelength range was set from 500 to 4,000 cm^-1^, and the scanning rate was 4 cm^−1^.

#### 2.4.7 Immunofluorescence staining

The PET/PDA/dECM carriers were fixed with 4% paraformaldehyde (PFA) at room temperature for 15 min, followed by two washes with PBS and then blocking. The primary antibodies (Affinity, United States) were diluted in 1% BSA in PBST as follows: COL I (1:200), COL IV (1:200), FN (1:200), LN (1:200), ELN (1:200). The diluted primary antibodies were incubated with the samples in the dark for 1 h. Subsequently, secondary antibodies (diluted at a ratio of 1:200 in 1% BSA in PBST, Affinity, United States) were incubated for 1 h in the dark. The carriers were observed using a laser scanning confocal microscopy (TCS SP5, Leica, Germany) with a scanning depth of approximately 200 μm and a layer thickness of 10 μm.

### 2.5 Cell experiments

#### 2.5.1 Cytotoxicity assessment of PET composite carrier

Following the preparation standards of the document “GBT 16886.12–2017,” an appropriate amount of PET/PDA/dECM carrier was immersed in complete culture medium at a ratio of 0.1 g/mL. The carrier was then incubated at 37°C in a CO_2_ incubator for 24 h and subsequently filtered for sterilization. 3T3 cells were seeded at a density of 5×10^3^ cells per well, and after 24 h, the culture medium was replaced with the extraction medium for another day. The MTT assay was used to assess cytotoxicity.

Sterilized PET/PDA/dECM carriers were placed in 24-well plates, and 3T3 cell suspension was seeded on the carriers at a density of 2×10^5^ cells/200 μL. The cells were cultured until the 3rd and 5th day, after which the culture medium was removed, and the carriers were rinsed with PBS. Then, 0.5 mL of fluorescein diacetate (FDA, Sigma, United States) working solution was added to stain the cells for approximately 5 min. The staining solution was removed, and the cells were rinsed twice with PBS. The distribution of cells was observed using a fluorescence microscope (IX71, Olympus, Japan).

#### 2.5.2 Effect of PET composite carrier on the *In vitro* proliferation of HUCMSCs

Passages six to eight of HUCMSCs (Guangzhou Saliai Stem Cell Science and Technology Co., Ltd., China) were seeded at a density of 5×10^3^ cells/cm^2^ in a 6-well plate as the control group. PET/PDA and PET/PDA/dECM carriers were subjected to Co60 gamma irradiation for sterilization (25 kGy) before cell seeding. The carriers were then placed in 24-well plates and seeded with HUCMSCs at a density of 1 × 10^5^ cells per carrier. The cells were continuously cultured in a cell incubator for 14 days, with fresh culture medium replaced every 2 days. Cells were digested using 0.1% type I collagenase for 1 h and 0.25% trypsin for 5 min. The cell proliferation rate at specific time points (Day 1, 3, 5, 7, 10, 14) was calculated to reflect cell proliferation activity.

#### 2.5.3 Effect of PET composite carrier on the adhesion and morphology of HUCMSCs

On the 7th and 14th day of cell culture, carriers were removed, rinsed with PBS, and stained with an appropriate amount of FDA (Sigma, United States) staining solution. The cells were then observed using a fluorescence microscope (IX71, Olympus, Japan). Cell cytoskeleton staining was performed using a cell staining kit (Merck millipore, Germany), and observations were made using a confocal microscope (TCS SP5, Leica, Germany). Furthermore, after fixing and freeze-drying the samples, they were sputter-coated with gold and placed in a vacuum chamber with a vacuum degree of 3 × 10^−3^ kPa. The acceleration voltage and current were set to 20 kV and 10 mA, respectively. The samples were observed under a scanning electron microscope (Quanta 400FEG, FEI, United States).

#### 2.5.4 Immunophenotypic analysis of HUCMSCs

At Day 7 and Day 14 of culture, cells were digested with 0.1% type I collagenase for 1 h and 5 min with trypsin to collect the cells. The single-cell suspension was filtered through a cell strainer. The cells were then incubated with the following fluorescence-conjugated primary antibodies: CD73-PE, CD105-FITC, CD90-APC, CD34-APC, CD11b-PE, CD45-PE, CD19-PC7, and HLA-DR-PC7 (BD Biosciences, United States of America) at 4°C in the dark for 30 min. Subsequently, flow cytometry (FACSCalibur, Becton Dickinson, United States) was used for detection, and isotype-matched unlabeled monoclonal antibodies were used as background controls. FlowJo software was employed for image processing.

#### 2.5.5 qPCR analysis of cytokine expression in HUCMSCs

At Day 7 and Day 14, RNA from different groups of HUCMSCs was extracted using an RNA extraction kit (Invitrogen, United States). Reverse transcription was performed using the HiScript^®^ II Q Select RT SuperMix for qPCR kit (Vazyme, United States) to obtain cDNA. The expression levels of HGF, TGF-β, VEGF, IL-10, KGF, and Ang-1 were detected using RT-PCR. GAPDH was used as the reference gene. The relative expression levels of mRNA were calculated using the following formulas:

$$\text{Relative Quantification }\left(\text{RQ}\right)=2^{-△△C_{T}},$$ (2)

$$\text{Target Gene }{△C}_{T}=\text{Target Gene}{△C}_{T}-\;\text{Reference Gene}{△C}_{T},$$ (3)

$$△{△C}_{T}=Treatment\;Group\;{△C}_{T}\;-\;Control\;Group\;{△C}_{T}.$$ (4)

#### 2.5.6 Western blot analysis of cytokine expression in HUCMSCs

At Day 7 and Day 14, Western blot analysis was conducted to examine the protein expression of cytokines. The protein content was quantified using the BCA assay kit (KeyGEN, Jiangsu, China). 25 μg of denatured protein was loaded onto a 5% stacking gel and run at 60V for 25 min, followed by separation on a 12% resolving gel at 120 V for 1 h. The proteins were then transferred to a PVDF membrane, blocked, and incubated with the primary antibodies (rabbit-derived antibodies against Laminin, Elastin, Fibronectin, Collagen I, Collagen IV, and Fibronectin, Affinity, United States) at a 1:500 dilution in 1% BSA in PBS. Afterward, the membrane was incubated with secondary antibodies (goat anti-rabbit Fluor488-conjugated secondary antibodies, Affinity, United States) at a 1:5000 dilution in 1% BSA in PBS for 1 h. The membrane was washed 3–5 times and subjected to exposure, development, and fixation using an ECL chemiluminescence kit (KeyGEN, Jiangsu, China). The results were analyzed using ImageJ software.

#### 2.5.7 ELISA analysis of cytokine secretion in HUCMSCs

On Days 1, 3, 5, 7, 9, 11, and 13, cell culture supernatants were collected and stored at −80°C. ELISA kits (Elabscience, China) were used to measure the concentrations of HGF and TGF-β1 in the supernatants.

### 2.6 Statistical analysis

All data in this study are presented as mean ± standard deviation (*n* = 3). One-way analysis of variance (ANOVA) was used to compare differences between different groups, and a significance level of *p* < 0.05 was considered to indicate significant differences in the data.

## 3 Results

### 3.1 Morphology of decellularized and enzymatically digested tissue

The decellularization of animal tissues aims to remove cellular components that may trigger immune reactions while preserving the essential constituents of the extracellular matrix (ECM), such as collagen, glycosaminoglycans, and bioactive factors. Based on observed *in vivo* remodeling responses and results to avoid cellular and adverse host reactions, the following criteria have been established for assessing the effectiveness of decellularization (Crapo et al., 2011): 1) Dry weight <50 ng/mg DNA; 2) DNA fragments <200 bp; 3) Absence of nuclear material as evidenced by DAPI or H&E staining. As shown in Figure 1A, the freshly harvested porcine lung tissue appears bright red, but after the decellularization process, it turned into a milky-white color, indicating the successful removal of surface cells. The enzymatically digested tissue appears as a milky-white, viscous fluid containing numerous bioactive ECM components, which can be utilized for subsequent material coating purposes.

### 3.2 Characterization of decellularization effect

During the decellularization process, residual nuclear components can cause non-specific cellular reactions and immune rejection responses. Therefore, cellular constituents should be thoroughly removed whenever possible. In this study, DAPI staining was employed to visualize lung tissue slices before and after decellularization, as shown in Figure 1C. In the original lung tissue, numerous blue circular nuclei can be observed, while after decellularization, hardly any blue spots are visible. The distribution of nuclear matter in the decellularized solution appears more uniform, and the presence of blue nuclei is almost negligible.

H&E staining utilizes eosin and hematoxylin dyes to visualize tissue structures and cell distribution. As depicted in Figure 1D, the lung tissue exhibits a reticular structure with the alveoli forming pores, separated by a layer of connective tissue (alveolar septum). In the original lung tissue, a large number of purple nuclei are embedded in the red reticular matrix, while after decellularization, hardly any purple nuclei are found.

Hoechst 33258 exhibits high affinity binding to the major groove of double-stranded DNA but has weak reactivity towards RNA. Therefore, this study employed Hoechst 33258 dye to stain double-stranded DNA in the tissue, enabling quantitative analysis of DNA. As shown in Figure 1E, the total DNA content in the tissue before decellularization was 14.51 ± 0.79 μg/mg dry weight of tissue, while after decellularization, it reduced significantly to 36.70 ± 3.62 ng/mg dry weight of tissue.

Glycosaminoglycans (GAGs) play a crucial role in maintaining the stability of collagen and elastin in the extracellular matrix (ECM) (Linhardt and Toida, 2004) and are essential for ECM assembly and remodeling during homeostasis and tissue repair processes *in vivo* (Wang et al., 2021). The original lung tissue had a polysaccharide content of 20.91 ± 1.37 μg/mg dry weight of tissue, which reduced to 5.04 ± 0.31 μg/mg dry weight of tissue after decellularization (Figure 1E).

Collagen is the most abundant component in the ECM, accounting for over 30% of its composition. It maintains the structural strength and stability of the extracellular environment and plays a crucial role in cell growth processes (He et al., 2013). The original lung tissue had a collagen content of 313.89 ± 6.36 μg/mg dry weight of tissue, whereas after decellularization, the collagen content increased to 383.68 ± 54.71 μg/mg dry weight of tissue.

### 3.3 Characterization of PET composite carrier

**FIGURE 2:**
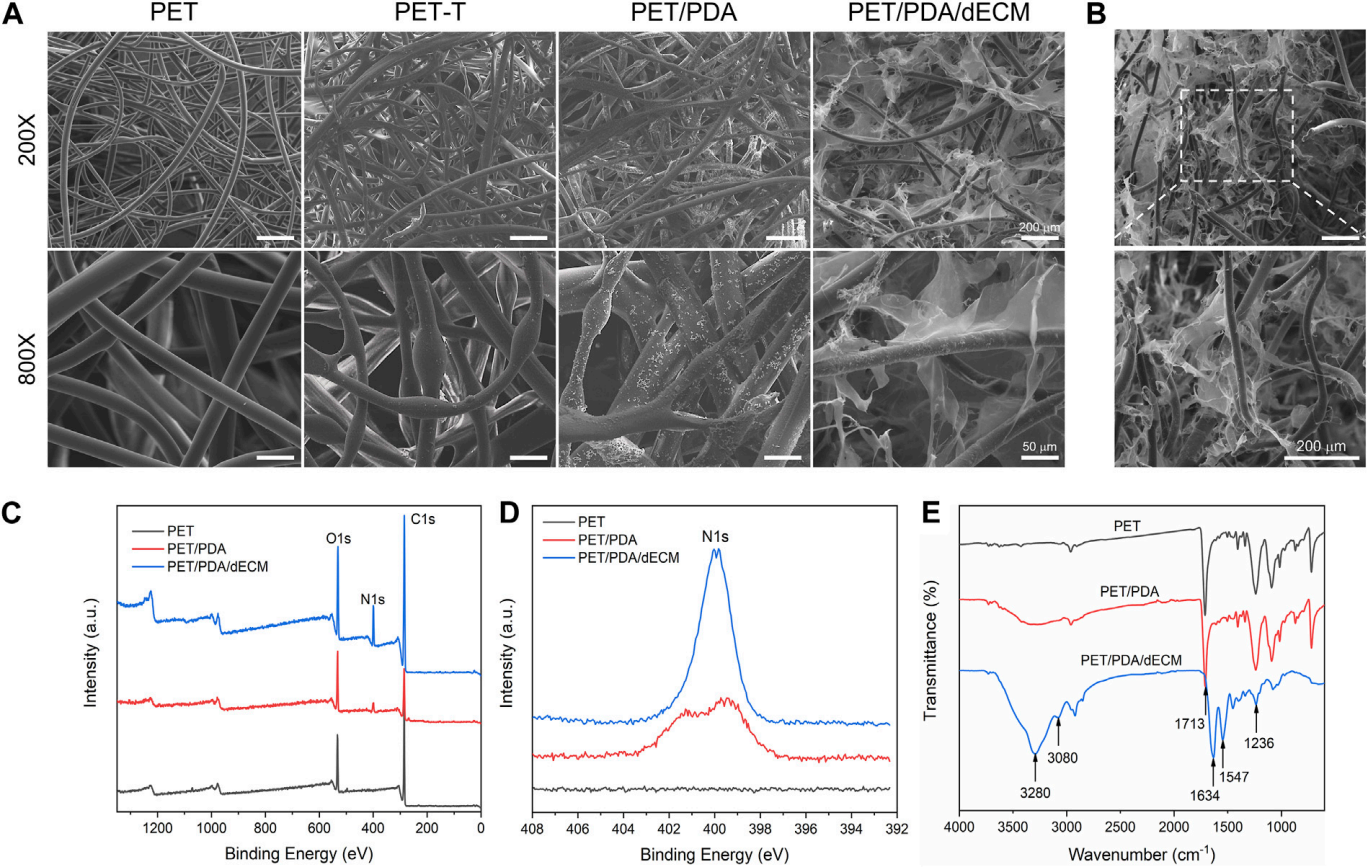
Characterization of PET carriers after different treatments. **(A)** Scanning electron microscope images of PET fibers with different surface modifications. **(B)** Cross-sectional view of PET/PDA/dECM scaffold. **(C)** X-ray photoelectron spectroscopy (XPS) full-spectrum scan results of PET, PET/PDA, and PET/PDA/dECM scaffolds. **(D)** High-resolution scan of the N1s peak in the XPS results of PET, PET/PDA, and PET/PDA/dECM scaffolds. **(E)** Fourier-transform infrared spectroscopy (FTIR) results of PET, PET/PDA, and PET/PDA/dECM scaffolds. Scale bars in **(A)** represent 200 μm and 50 μm and in **(B)** represent 200 μm.

The surface microstructure of the PET carrier was observed and analyzed using SEM, and the results are shown in Figure 2A. The untreated PET fibers exhibited a disorderly arrangement, forming a three-dimensional porous network with interwoven fibers and uniform diameters. The surface was smooth without nodes. After the thermal treatment at 140°C for 4 h, the surface of the PET fibers became rough, possibly due to the occurrence of heat-induced crystallization in PET (Hill et al., 2015), but the overall structure of the carrier remained unchanged. After coating with the decellularized extracellular matrix and freeze-drying, the decellularized extracellular matrix formed a thin film-like structure evenly distributed on the fibers, filling the large pores of the PET fibers. From the cross-sectional view of the PET/PDA/dECM carrier (Figure 2B), it can be observed that the extracellular matrix not only exists on the surface of the carrier but also penetrates into the interior, intertwining with the PET fibers to form an interpenetrating network structure.

To characterize the grafting of the carrier, XPS analysis was performed on different carriers in this study. Figures 2C, D show the full-wavelength scan and high-resolution scan of the N1s peak for PET before and after modification. Generally, the molecular structure of PET contains only three elements: C, H, and O. From the graph, it can be observed that all three curves of PET, PET/PDA, and PET/PDA/dECM exhibit characteristic peaks of C1s (284.8 eV) and O1s (531.3 eV). After grafting polydopamine, the PET/PDA curve exhibits an absorption peak of N1s at a binding energy of 399.7 eV (Ren et al., 2019), with a relative nitrogen content of 7.31% (Table 1), indicating a substantial amount of polydopamine grafting. Upon the introduction of decellularized extracellular matrix, the N1s peak in the PET/PDA/dECM curve significantly differs from that of PET/PDA. The nitrogen content in the PET/PDA/dECM carrier notably increases to 10.93%, which is consistent with the findings of Topuz and Uyar, (2017). The incorporation of proteinaceous substances through grafting leads to a significant increase in nitrogen content.

**TABLE 1:** Atomic percentages of PET, PET/PDA and PET/PDA/dECM carriers.

Sample names	C (%)	O (%)	N (%)
PET	79.35	18.75	0
PET/PDA	66.28	22.04	7.31
PET/PDA/dECM	71.06	16.97	10.93

To characterize the chemical structure of the carriers, this study conducted FTIR analysis on carriers grafted with polydopamine and decellularized extracellular matrix (dECM). As shown in Figure 2E, the PET curve exhibits absorption peaks at 1713 cm^-1^ and 1016 cm^-1^, corresponding to the C=O and C-O stretching vibrations of the PET molecular structure (Fávaro et al., 2007). The absorption band at 721 cm^−1^ is due to the vibration of hydrogen in the para position of the benzene ring in PET structure (Parvinzadeh et al., 2010). The peak at 1248 cm^−1^ represents the ester group vibration, while the absorption band around 2,700–3,000 cm^−1^ is attributed to the -CH stretching vibrations in saturated carbons. In the PET/PDA curve, a strong broad peak appears in the range of 3,000–3,500 cm^−1^, mainly caused by O-H and N-H bonds, indicating successful grafting of polydopamine. The peaks at 3,280 cm^−1^ and 3,080 cm^−1^ represent the stretching vibrations of N-H and O-H, respectively. In the PET/PDA/dECM curve, the characteristic peaks at 1634 cm^-1^, 1547 cm^−1^, and 1236 cm^−1^ are attributed to the stretching vibrations of C = O, bending vibrations of N-H, and absorption of C-N (Lee et al., 2018; Choi et al., 2021). These peaks correspond to the amide I, amide II, and amide III bands, which are typical features of peptide-like substances, indicating the presence of extracellular matrix proteins.

**FIGURE 3:**
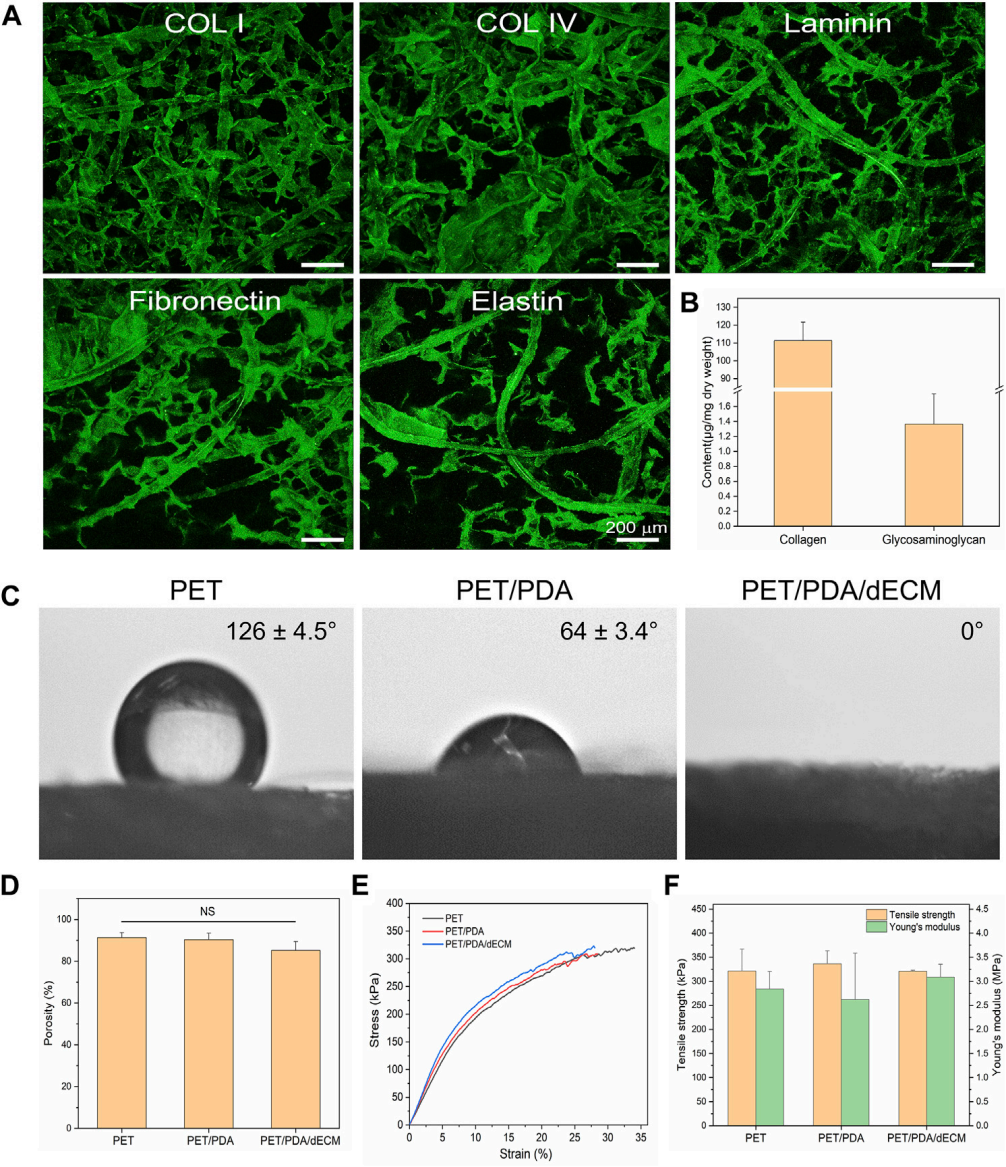
Characterization of PET carriers after different treatments. **(A)** Immunofluorescence staining of PET/PDA/dECM carriers (collagen I, collagen IV, laminin, fibronectin, elastin). **(B)** Collagen and polysaccharide content of PET/PDA/dECM carriers. **(C)** Surface static water contact angles of PET, PET/PDA, and PET/PDA/dECM carriers. **(D)** Porosity of PET, PET/PDA, and PET/PDA/dECM carriers. **(E)** Stress-strain curves of PET, PET/PDA, and PET/PDA/dECM carriers. **(F)** Tensile strength and Young’s modulus of PET, PET/PDA, and PET/PDA/dECM carriers. Scale bars in **(A)** represent 200 μm (data = mean ± SD; *n* = 3; NS represent not significant).

In order to observe the distribution of decellularized extracellular matrix (dECM) on the fiber carrier, this study performed immunofluorescence staining on the prepared PET/PDA/dECM carrier for common ECM proteins in lung tissue (collagen I, collagen IV, laminin, fibronectin, elastin). The results are shown in Figure 3A, where the green fluorescence of all five ECM proteins can be clearly detected on the PET/PDA/dECM carrier. The ECM proteins are attached to the fibers, forming a uniform coating on the fiber surface and continuous protein layers between the fibers. Furthermore, this study accurately quantified the content of collagen and glycosaminoglycan (GAG) on the PET/PDA/dECM carrier, as shown in Figure 3B, where the collagen content is 111.29 ± 10.38 μg/mg carrier dry weight, and the GAGs content is 1.36 ± 0.40 μg/mg carrier dry weight. Combined with the scanning electron microscopy results, most of the extracellular matrix is retained, significantly improving the carrier’s biocompatibility and promoting cell adhesion and interactions with the external environment.

In this study, a static water contact angle measurement instrument was used to detect the surface hydrophobicity of the materials. As shown in Figure 3C, pure PET exhibits strong hydrophobicity, which is unfavorable for cell adhesion and proliferation, as indicated by a water contact angle of 126.1° ± 4.5°. After grafting PDA, the hydrophilic groups in polydopamine significantly reduce the contact angle to 64.1° ± 3.4°, leading to a significant improvement in material hydrophilicity. When dECM is further introduced, the carrier exhibits complete hydrophilicity, with water droplets promptly spreading uniformly across the material surface, resulting in an exceedingly close-to-0° contact angle.

As shown in Figure 3D, the commercially available PET fiber sponge possesses a highly porous structure with a porosity of 91.3% ± 2.5%. Thermal treatment has minimal influence on the porosity of the sponge, and after grafting PDA, the porosity of the carrier is 90.3% ± 3.1%. After further coating with dECM, the porosity of the carrier is 85.2% ± 4.2%, showing a slight decrease but no significant difference from PET. This is mainly because dECM forms a thin film-like structure between the fibers, partially filling the porous structure of the carrier without altering its macroscopic structure.

As shown in Figure 3F, the tensile strength and Young’s modulus of the thermally treated PET sponge are 321.3 ± 45.6 kPa and 2.84 ± 0.36 MPa, respectively. As PDA is adsorbed in a granular form on the fiber surface without altering the fiber structure, the mechanical properties of the PET/PDA carrier remain largely unchanged, with a tensile strength of 336.3 ± 27.3 kPa and a Young’s modulus of 2.62 ± 0.97 MPa. The PET/PDA/dECM carrier exhibits a tensile strength and Young’s modulus of 321.0 ± 1.8 kPa and 3.09 ± 0.26 MPa, respectively, with a slight increase in Young’s modulus.

### 3.4 Effects of PET composite carrier on HUCMSCs proliferation and adhesion

**FIGURE 4:**
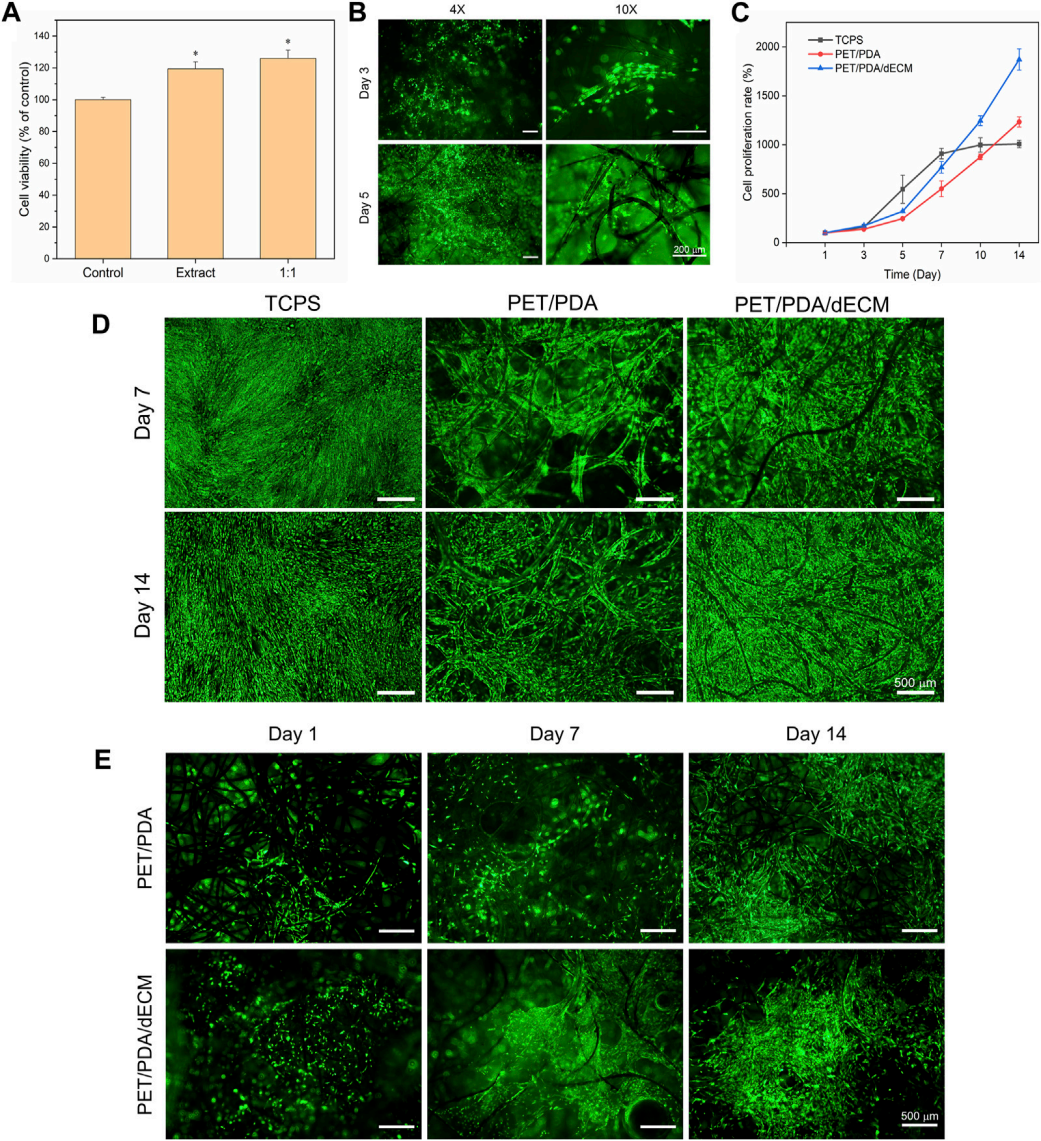
Effect of PET composite carriers on HUCMSCs proliferation and adhesion. **(A)** Cytotoxicity of PET/PDA/dECM carrier extract on 3T3 cells. The control group was utilized as a reference, where the cellular viability in the control group was set as 100%. **(B)** Fluorescence staining images of 3T3 cells cultured on PET/PDA/dECM carriers for 3 and 5 days. **(C)** Proliferation rate of HUCMSCs on TCPS, PET/PDA, and PET/PDA/dECM after 14 days of culture, with counting time points at Day 1, 3, 5, 7, 10, and 14. **(D)** Fluorescence staining images of HUCMSCs cultured on TCPS, PET/PDA, and PET/PDA/dECM carriers for 7 and 14 days. **(E)** Fluorescence staining images of HUCMSCs on the backside of PET/PDA and PET/PDA/dECM carriers after 1, 7, and 14 days of culture. Scale bars in **(B)** represent 200 μm and in **(D)** and **(E)** represent 500 μm (data = mean ± SD; *n* = 3; *p < 0.05).

First, the toxicity of the extracts from the PET/PDA/dECM carrier on 3T3 cells was evaluated using the MTT assay. As shown in Figure 4A, the viability of 3T3 cells cultured with the extract was higher than that of cells cultured in normal culture medium. Furthermore, when the extract was mixed with normal culture medium in a 1:1 ratio, the cell viability further increased. Additionally, this study seeded 3T3 cells onto the carrier to observe cell growth. As depicted in Figure 4A, a large number of viable cells were distributed on the carrier’s surface and inside, adhering to the fibers and extending along their direction, while gathering and growing at the intersections of fibers. Moreover, a thin film of dECM was observed between the fibers, with some cells adhering to the film and exhibiting elongated and stretched morphology. This is mainly attributed to the interaction between cell surface integrins and ECM bioactive substances, leading to the rearrangement of their own fibrous protein skeleton. It demonstrates the interaction between dECM and cells, promoting cell adhesion and spreading.

In this study, the cell proliferation rate was directly measured using cell counting, and the results at each time point were normalized to the cell quantity on the first day. Moreover, digestion of cells on the three-dimensional carrier with 0.1% type I collagenase and 0.25% trypsin effectively avoided errors caused by some cells not being fully digested. The cell proliferation rate results are presented in Figure 4C. In the first 7 days of culture, the cell proliferation rate in the two-dimensional well plate was higher than that in the PET/PDA and PET/PDA/dECM groups. This is because a greater number of cells adhered to TCPS surface, leading to higher cell attachment and survival rates and more cell-cell contacts, resulting in faster growth. In contrast, in the PET/PDA and PET/PDA/dECM groups, many cells leaked out of the pores of the three-dimensional carriers, resulting in a lower initial cell density, and cells were relatively isolated in a three-dimensional space, leading to slower proliferation. After 7 days of culture, due to the limited growth area of TCPS, the cells entered a contact inhibition state, and the cell number no longer increased, reaching about 9.1-fold. However, in the three-dimensional carrier groups, the cells continued to proliferate rapidly, with the proliferation rate surpassing that of the TCPS group around day 10. By day 14, the cell proliferation rate on PET/PDA carriers was approximately 12.3-fold, while on PET/PDA/dECM carriers, it was as high as 18.7-fold, 1.9 times higher than that of the TCPS group.

The vitality and distribution of HUCMSCs on TCPS and PET three-dimensional carriers were detected using FDA staining. As shown in Figure 4D, all three substrate groups exhibited relatively high cell viability. By the 7th day of culture, TCPS was mostly covered with cells, entering a state of contact inhibition. Cells on the PET/PDA carrier displayed an elongated spindle shape and grew along the direction of the fibers. On the PET/PDA/dECM carrier, cells were in contact with each other and formed contiguous growth at the intersections of the fibers, with a higher number of viable cells than the PET/PDA group. By day 14, the cell quantity and status on TCPS were not as favorable as on the 7th day, possibly due to prolonged contact inhibition leading to cellular aging (Cristofalo and Pignolo, 1993), resulting in changes in cell morphology. In contrast, cells on the PET/PDA carrier and PET/PDA/dECM group significantly increased compared to day 7, especially on the surface of the PET/PDA/dECM carrier, where cells almost filled the surface, while the interior of the carrier still provided growth space. These results were consistent with the cell proliferation rate results.

Furthermore, this study demonstrated through cell fluorescence staining on the backside of the carrier (Figure 4E) that a small number of cells were carried through the carrier and adhered to the fibers on the backside of the PET/PDA carrier.

**FIGURE 5:**
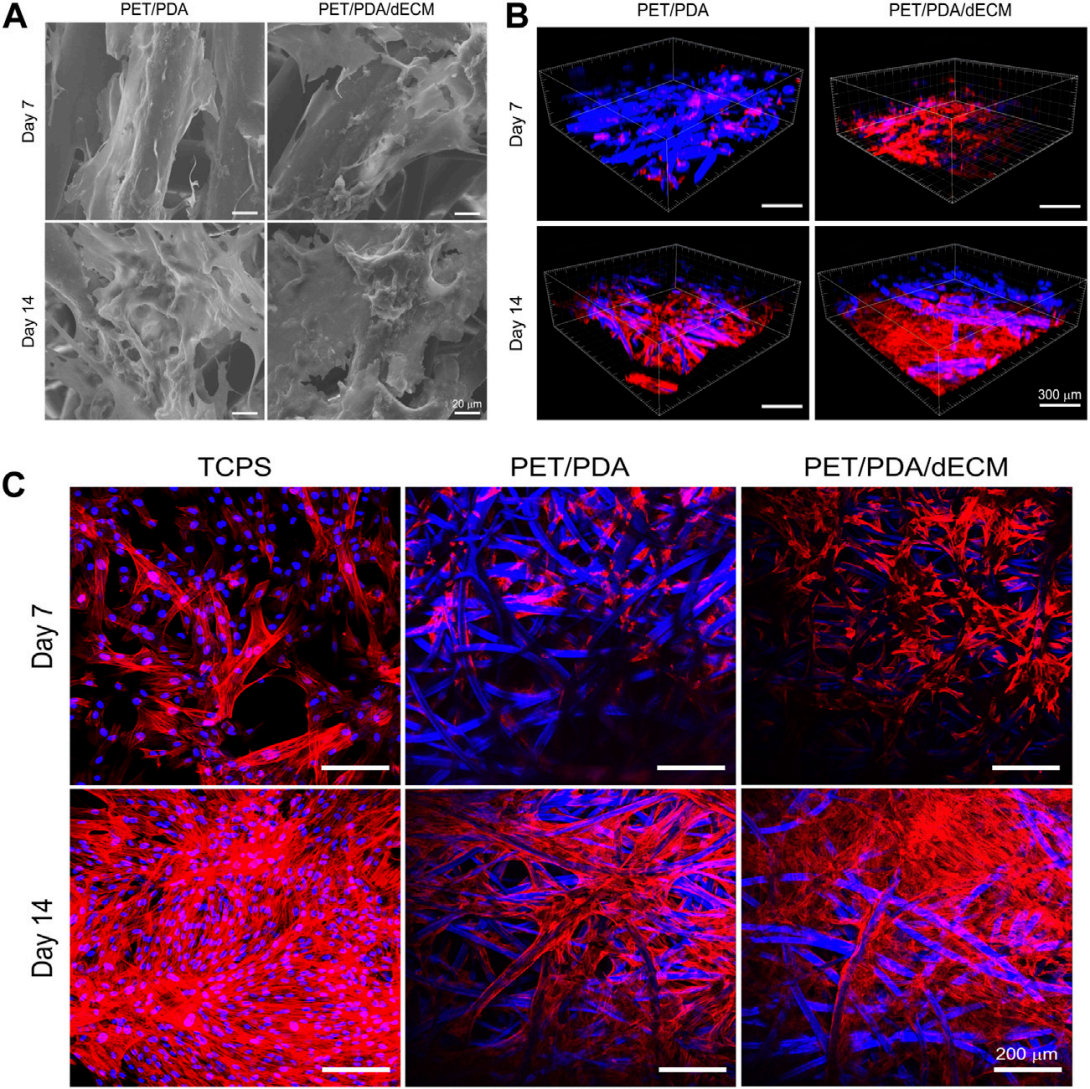
**(A)** Scanning electron microscopy images of HUCMSCs cells cultured on PET/PDA and PET/PDA/dECM for 7 and 14 days. **(B)** Three-dimensional scanning images of the cell cytoskeleton staining of HUCMSCs cultured on PET/PDA and PET/PDA/dECM for 7 and 14 days. Red represents actin filaments, blue spots indicate cell nuclei. The blue fibers in the carriers are due to the autofluorescence of PET under ultraviolet excitation. **(C)** Cell cytoskeleton staining of HUCMSCs cultured on TCPS, PET/PDA, and PET/PDA/dECM for 7 and 14 days. Scale bars in **(A)** represent 20 μm, in **(B)** represent 300 μm and in **(C)** represent 200 μm.

After cell seeding onto the material, surface transmembrane receptors such as integrins recognize adhesive sites, and cells continuously secrete ECM to enhance their interaction with the environment, promoting their own adhesion and growth. Scanning electron microscopy observation (Figure 5A) revealed that cells adhered and spread well on the fibers, secreting a significant amount of ECM to form a protein network, especially after 14 days of culture. The ECM secreted by the cells had interconnected, filling the gaps between PET fibers. Moreover, there was slightly more ECM deposition on PET/PDA/dECM compared to PET/PDA, which facilitated the extracellular matrix secretion of HUCMSCs.

Stem cells form transmembrane adhesive complexes with material surfaces through the interaction of adhesive molecules with active sites. These complexes are closely associated with actin-myosin cytoskeleton. Actin receives mechanical feedback from the external environment, leading to cytoskeletal rearrangement and changes in stem cell shape. Cell cytoskeleton staining was performed to observe the spreading morphology of cells on the materials and the arrangement of actin filament cytoskeleton. The results in Figure 5C showed that on day 7, the TCPS group displayed well-constructed cytoskeleton with good cell spreading and neighboring cells interconnected through intercellular filaments. In the PET/PDA group, cells clustered at fiber intersections, and actin filaments distributed along the fibers. Due to uneven seeding, some areas of cells remained isolated and failed to establish close contacts. On PET/PDA/dECM, the cell cytoskeleton network was notably denser than in the PET/PDA group, with actin filaments not only distributed along the fibers but also stretching between them, displaying a good tensile state. After 14 days of culture, with a significant increase in cell number, the actin filament cytoskeleton on all three substrates became denser. The TCPS surface was almost filled with actin filaments, leaving no extra growth space for cells. On PET/PDA, actin filaments formed patches at fiber intersections, indicating more secretion on the surface, and the internal space extended along the fiber direction. The surface of PET/PDA/dECM was covered with actin filaments. Figure 5B showed three-dimensional scanning images of cell cytoskeleton staining, providing a more intuitive observation of cell growth on the substrate along the surface and longitudinally, with cytoskeleton extending and expanding between fibers. After 14 days of culture, cells were in close contact, tending to grow in sheets, especially on PET/PDA/dECM, where large areas of cell layers were observed.

### 3.5 Immunophenotype analysis

During long-term two-dimensional (2D) culture *in vitro*, MSCs gradually lose pluripotency, and surface-specific antigens undergo changes. However, three-dimensional (3D) culture is conducive to maintaining the phenotype of stem cells. The surface antigen expression of HUCMSCs cultured on 2D surfaces and 3D carriers was assessed using flow cytometry. According to the International Society for Cell and Gene Therapy (ISCT) standards for identifying MSCs, MSCs should express CD90, CD73, and CD105 antigens, and should not express or show low expression of CD34, CD14 or CD11b, CD45, CD79 or CD19, and HLA-DR surface antigens.

CD73 is an extracellular 5′-nucleotidase widely expressed on the surfaces of lymphocytes and endothelial cells and is a critical factor in the conversion of pro-inflammatory adenosine triphosphate (ATP) to anti-inflammatory adenosine metabolism (Ode et al., 2011). CD90, also known as Thy-1, is a glycosylphosphatidylinositol-anchored glycoprotein associated with the undifferentiated state of MSCs. Its reduced expression is correlated with enhanced osteogenic and adipogenic differentiation of MSCs *in vitro* (Moraes et al., 2016). CD105 is distributed on the cell surface and is a component of the TGF-β receptor. CD45 is a pan-leukocyte marker, CD34 marks endothelial cells and hematopoietic progenitor cells, CD19 is a marker for B lymphocytes, and HLA-DR is expressed in macrophages, B cells, and activated T cells.

Table 2 shows the expression levels of various antigens. When cultured for 7 days, cells in both the TCPS and PET/PDA/dECM groups exhibited positive marker expression and no negative marker expression, indicating that HUCMSCs maintained a good growth state and did not undergo differentiation during the 7-day culture on TCPS and PET/PDA/dECM. However, the CD105 expression level in the PET/PDA group was only 45.2%, which did not meet the requirements. When cultured for 14 days, it was found that all antigens expressed by cells in the PET/PDA/dECM group met the standard criteria. In the TCPS group, all antigens were expressed normally except for CD105, which had an expression level of 24.1%. In the PET/PDA group, the expression of CD105 was 17.5%, and there was a slight upregulation in the expression of CD34 (3.4%) and HLA-DR (3.3%).

**TABLE 2:** The expression levels of surface specific antigens on HUCMSCs cultured on different substrates for 7 and 14 days.

Marker	TCPS (%)		PET/PDA (%)		PET/PDA/dECM (%)	
	Day 7	Day 14	Day 7	Day 14	Day 7	Day 14
CD73	100.0	100.0	99.9	100.0	100.0	100.0
CD90	100.0	100.0	100.0	100.0	100.0	100.0
CD105	99.5	24.1	45.2	17.5	94.5	98.8
CD11b	0	0	0	0	0	0
CD34	0	0.8	0	3.4	0.2	0
CD45	0	0	0	0	0	0
CD19	0	0	0	0	0	0
HLA-DR	0.1	1.3	0	3.3	0.5	0.4

### 3.6 Analysis of HUCMSCs cytokine gene expression and protein expression

The immunomodulatory functions of MSCs primarily rely on the paracrine mechanism, where they secrete various functional factors. In this study, we selected several common MSCs cytokines (HGF, TGF-β, Ang-1, IL-10, VEGF, KGF) that have been extensively studied and shown to play significant roles in regulating inflammation, anti-apoptosis, promoting angiogenesis, and tissue repair (Mei et al., 2007; Pianta et al., 2015; An et al., 2018).

**FIGURE 6:**
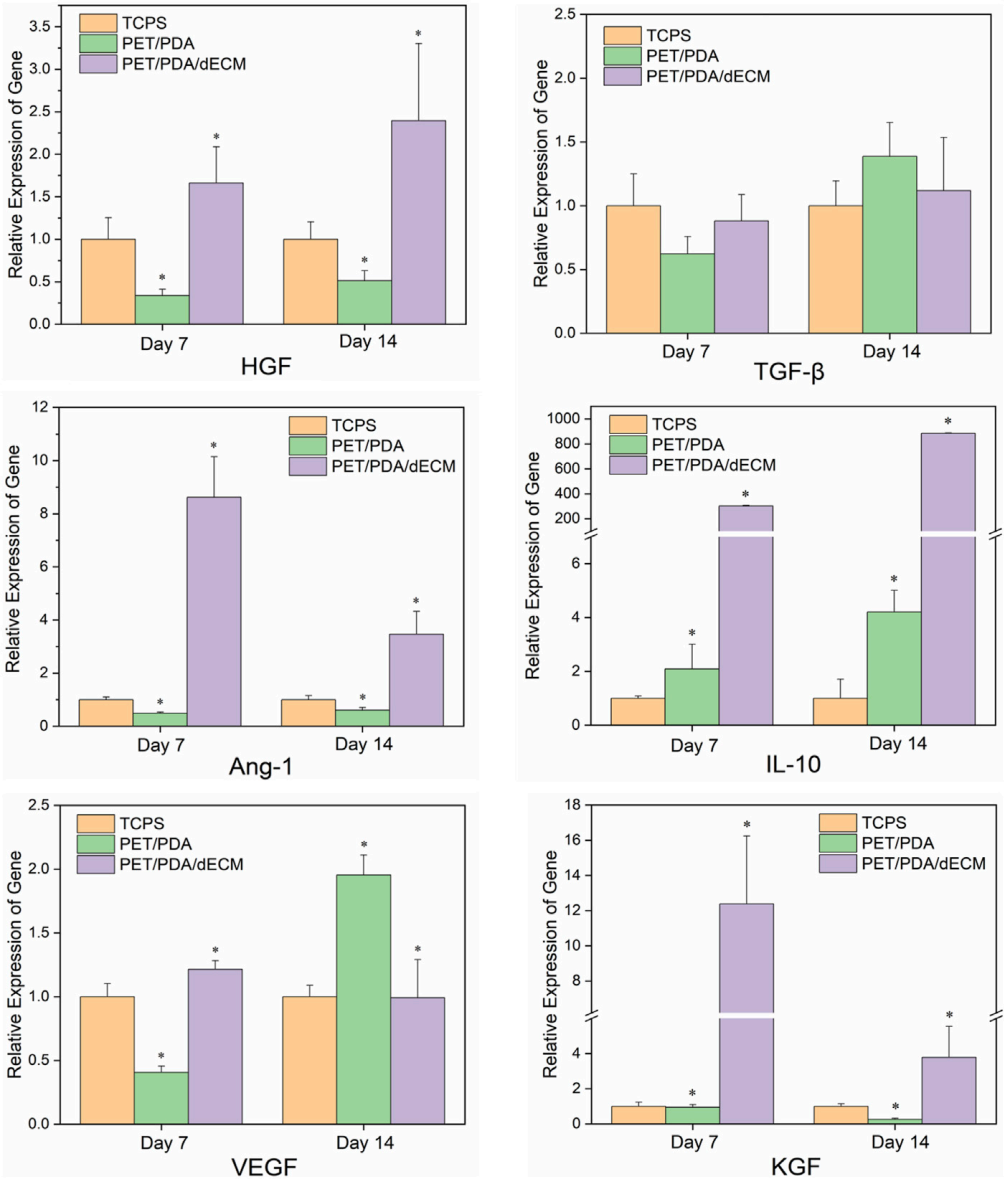
The relative gene expression of cytokines in HUCMSCs cultured on TCPS, PET/PDA, and PET/PDA/dECM carriers for 7 days and 14 days, respectively. The mRNA expression levels of each group were normalized against the TCPS on the same day (data = mean ± SD; *n* = 3; *p < 0.05).

The mRNA expression levels of the relevant cytokines are shown in Figure 6. Cells cultured on TCPS, PET/PDA, and PET/PDA/dECM substrates all exhibited gene expression of the selected factors. At both 7 and 14 days, the gene expression of relevant factors in the PET/PDA/dECM group was comparable to or significantly higher than that in the TCPS group, and, except for TGF-β and VEGF, all other factor expressions were significantly higher than in the PET/PDA group. At day 7, the expression of factors in the PET/PDA group was lower or comparable to the TCPS group, except for IL-10, which was higher than in the TCPS group. By day 14, the expression of factors in the PET/PDA group, except for TGF-β, VEGF, and IL-10, was lower than in the TCPS group.

**FIGURE 7:**
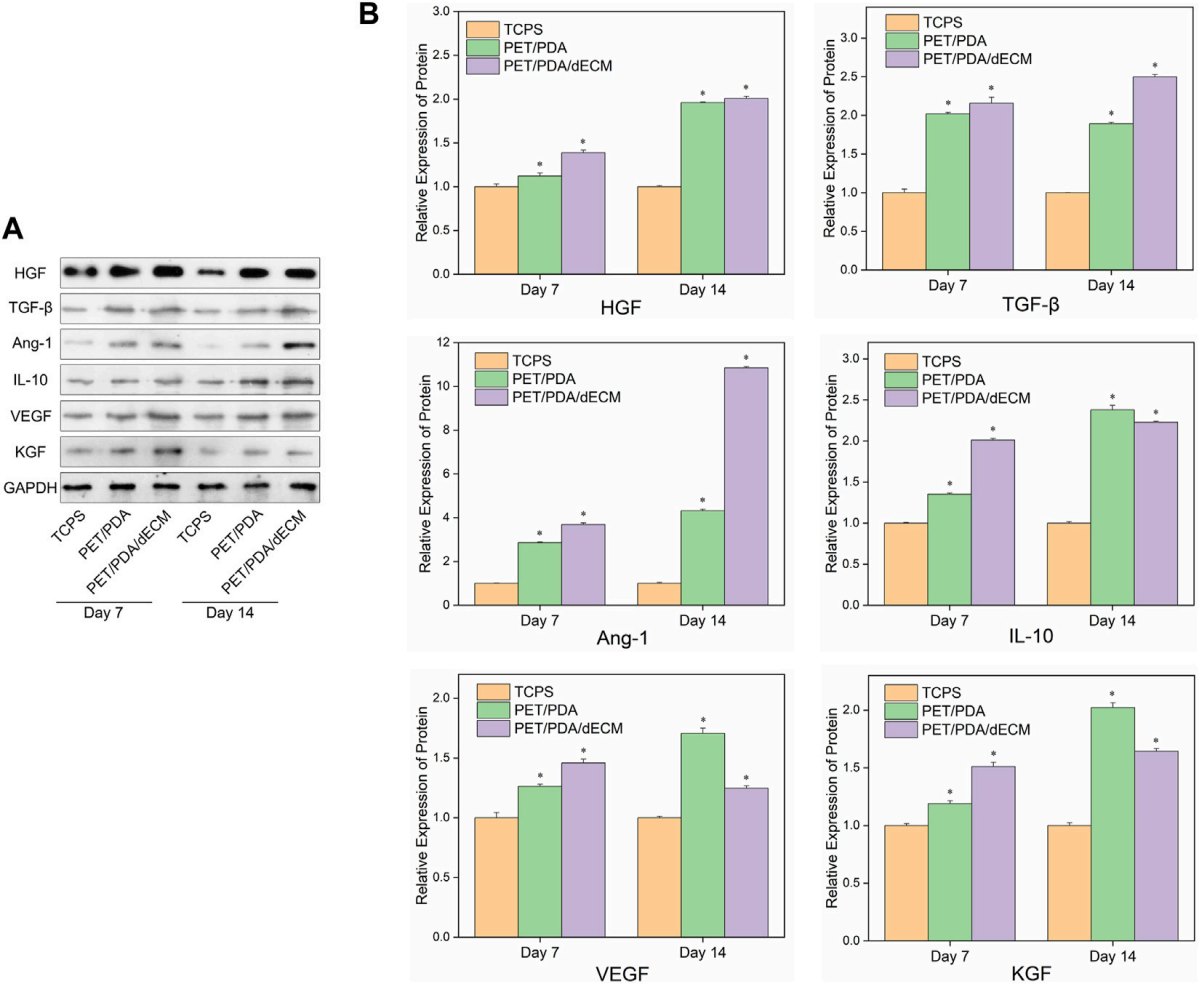
**(A)** Representative images of Western blot and **(B)** the relative protein levels of cytokines semi-quantified by ImageJ in HUCMSCs cultured on TCPS, PET/PDA and PET/PDA/dECM carriers for 7 days and 14 days, respectively. The protein expression levels of each group were normalized against the TCPS on the same day (data = mean ± SD; *n* = 3; *p < 0.05).

Figure 7 shows the Western blot protein band grayscale images and relative expression levels of active factors secreted by HUCMSCs cultured on different materials. At both 7 and 14 days, the expression levels of cytokine proteins in the PET/PDA and PET/PDA/dECM groups were significantly higher than in the TCPS group. At day 7, except for TGF-β expression, the PET/PDA/dECM group showed higher protein expression of other cytokines compared to the PET/PDA group. By day 14, except for IL-10, VEGF, and KGF, the protein expression of other factors in the PET/PDA group was lower than in the PET/PDA/dECM group.

### 3.7 Cytokine concentration determination

**FIGURE 8:**
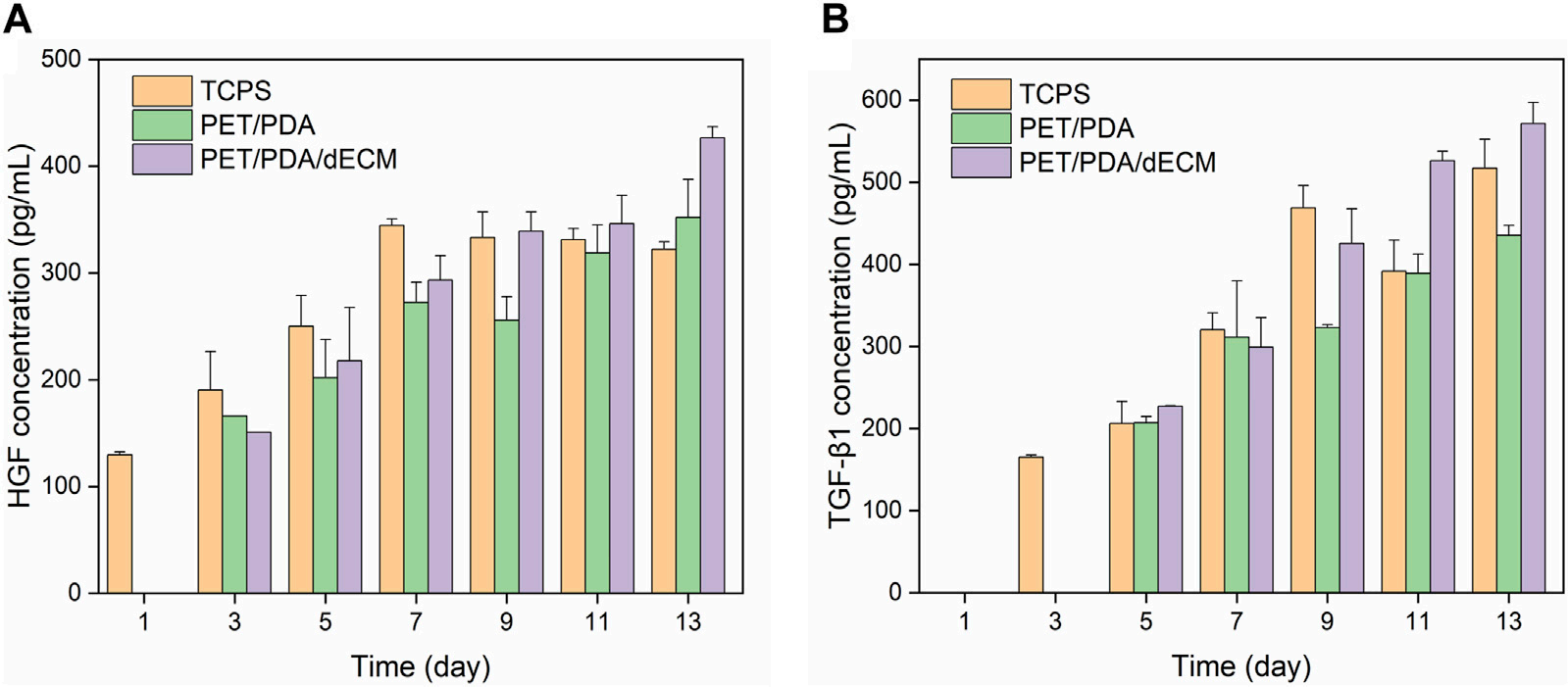
The production of **(A)** HGF and **(B)** TGF-β1 by HUCMSCs in cell culture supernatants on days 1, 3, 5, 7, 9, 11, and 13 (Data = mean ± SD; *n* = 3).

Active factors are transcribed as mRNA within cells, guiding the synthesis of proteins, which are ultimately secreted into the extracellular environment to play roles in immune regulation and promoting tissue repair (Fu et al., 2017). In this study, the concentrations of HGF and TGF-β1 in the cell culture supernatant were further measured. The results, as shown in Figure 8, demonstrate that compared to the PET/PDA and PET/PDA/dECM groups, cells cultured on TCPS secrete more HGF and TGF-β1 during the first 3 days, and this secretion continues to increase until day 9. At this point, the cells enter contact inhibition state, and the secretion of cytokines becomes stable. In the PET/PDA and PET/PDA/dECM groups, HGF secretion was not detected on day 1, and TGF-β1 secretion was not detected on days 1 and 3. This may be due to the fact that during seeding, a large number of cells leaked out of the fiber pores, resulting in a sparse cell population with cytokine secretion at a concentration too low to be detected. The lack of TGF-β1 detection on day 1 in the TCPS group is also attributed to the low cell population, resulting in cytokine secretion concentrations below the detection limit of the ELISA kit.

In the PET/PDA and PET/PDA/dECM groups, the secretion of HGF continuously increased from day 3 to day 13, reaching its maximum level on day 13, with concentrations of 352.1 ± 35.7 pg/mL and 426.6 ± 10.3 pg/mL, respectively. Moreover, in the PET/PDA/dECM group, the HGF level surpassed that of the TCPS group starting from day 9 (339.2 ± 18.1 pg/mL), and a similar trend was observed for TGF-β1 secretion. Additionally, at all time points, the secretion of HGF and TGF-β1 in the PET/PDA/dECM group was generally higher than that in the PET/PDA group.

## 4 Discussion

The relevant characterisation results demonstrate that subsequent to the application of the cellular decontamination methodology employed in this investigation, the vast majority of cellular components and DNA have been successfully eradicated. It is noteworthy that: the notable increase in collagen content after decellularization is mainly due to the high molecular weight of collagen, its intermolecular entanglement, and relatively low solubility, which contribute to its higher retention rate. Additionally, the removal of cellular and other components during the decellularization process leads to a higher proportion of collagen, which is consistent with the findings of Wu et al. (2015). These collagen components provide a wealth of active sites for subsequent cell culture, promoting cell adhesion, proliferation, and cytokine expression (Peng et al., 2017). Furthermore, the reduction in glycosaminoglycans can be attributed to the loss of water-soluble polysaccharides that dissolve in water during the decellularization process. However, a significant amount of glycosaminoglycans remains preserved, which is favorable for subsequent cell culture and functional expression. It underscores that the employed decellularization processing method is advantageous in significantly abating occurrences of non-specific cellular responses and immune rejection reactions. Concurrently, the preservation of essential elements such as polysaccharides and collagen proteins serves to greatly enhance cellular proliferation, tissue regeneration, and the manifestation of cellular functionalities.

The morphological and other geometric characteristics of PET substrates can exert influence on the adhesion, proliferation, and differentiation of Mesenchymal Stem Cells (MSCs) on their surfaces (Xiao et al., 2023). For instance, surface morphology, including features such as surface roughness and texture, can impact MSC adhesion, subsequently affecting their proliferation and differentiation (Tang et al., 2010; Song et al., 2022). The pore structure, on the other hand, provides a three-dimensional environment for cell adhesion and growth, mimicking the structure of native tissues. The pores must possess appropriate size, shape, and uniformity to serve as necessary conditions for cell growth and adhesion (Murphy et al., 2016). Additionally, the mechanical properties of the material, such as stiffness and elasticity, can modulate the differentiation of MSCs by influencing the biomechanical behavior of the cells (Engler et al., 2006). It is essential to emphasize that the material employed in this study is a commercially available, pre-commercialized PET sponge. PET materials with similar fibrous structures and subjected to surface modifications have been empirically proven to effectively support cell growth and achieve commercial viability. Therefore, this study did not undergo further optimization of the surface morphology or other geometric characteristics of the PET material. Instead, the chosen approach involved enhancing the material’s biocompatibility through surface modification and loading with decellularized extracellular matrix.

The characterization outcomes of the prepared carrier demonstrate that the synthesized substrate has effectively undergone grafting with PDA and dECM, exhibiting complete hydrophilicity, a high porosity rate, and commendable mechanical properties. Moreover, it is rich in a multitude of ECM proteins, showcasing exceptional biocompatibility. As per our previous research work (Ye et al., 2022), the thermal treatment of the PET fiber sponge further improved the structural strength, including mechanical properties and thermal stability, which is advantageous for maintaining the stability of the carrier during long-term cell culture experiments. After dopamine modification, polydopamine was self-polymerized on the surface of PET fibers, forming particles or particle aggregates adsorbed onto the fiber surface. Polydopamine contains various bioactive groups (such as ortho-diphenols, -OH, and -NH2), which can form strong bonds with the PET fiber surface, and during the grafting process, some ortho-diphenol groups of dopamine may be oxidized to quinones, which further participate in self-polymerization to form the PDA layer. Moreover, the residual ortho-diphenols on PDA can undergo Michael addition with some functional groups (such as -NH2 and -SH) in ECM proteins (Lee et al., 2009), thereby fixing the extracellular matrix molecules on the fibers, completing the modification, and ensuring a highly efficient and non-toxic reaction. Furthermore, the incorporation of dECM has significantly augmented the cellular compatibility of the carrier. This effectively fills the large inter-fiber pores and provides numerous active sites for cell adhesion, such as collagen and glycoproteins, promoting the interaction between cells and the substrate material. ECM can directly regulate cell functions through certain signaling pathways and can also control the expression of growth or differentiation factors, thus regulating cell proliferation and phenotype (Rosso et al., 2004).

The surface hydrophilicity of biomaterials influences cell adhesion, thereby regulating their secretion behavior and functional expression. Cell adhesion to materials depends on the interaction between surface adhesive molecules and active sites on the material surface (Ayala et al., 2011). The cell carrier fabricated in this study, post PDA and dECM modifications, exhibits a contact angle of 0°, manifesting absolute hydrophilicity. This characteristic will prove advantageous for the subsequent seeding and cultivation of cells. The pore size and porosity of three-dimensional biomaterial scaffolds directly influence their functionality. The fabricated microcarrier possesses a structure characterized by pronounced porosity and interconnectedness, which can effectively release proteins, genes, or cytokines, facilitate the diffusion of nutrients and oxygen, and transport metabolic waste, thereby enhancing cellular functional expression. Mechanical signals are an important way through which scaffold materials influence cells, guiding and coordinating cellular activities within tissues. Integrin proteins on the cell surface transmit signals to intracellular cascades, leading to cytoskeletal rearrangement and construction, thereby enabling adaptive transformation of cell morphology in response to mechanical signals at the material interface. This, in turn, regulates cellular functions such as adhesion, migration, proliferation, differentiation, and apoptosis. Compared to some natural materials, such as collagen sponge (tensile strength: 130.8 kPa, Young’s modulus: 9.3 kPa), collagen hydrogels (Hartwell et al., 2011) (Young’s modulus: 0.2 MPa), and hyaluronic acid hydrogels (Tavsanli and Okay, 2017) (tensile strength: 111 kPa), the mechanical performance of the PET/PDA/dECM carrier is more prominent, providing better structural support for cells during long-term cultivation.

The results obtained from co-culturing with cells further substantiate that PET/PDA and PET/PDA/dECM substrates exhibit negligible toxicity, showcasing exceptional cellular compatibility, promoting HUCMSCs’ adhesion, migration, and proliferation, facilitating interactions between cells and the external environment. In traditional two-dimensional culture, mesenchymal stem cells (MSCs) gradually lose their proliferation and differentiation potential over time. Three-dimensional culture is more favorable for the interaction between cells and their external environment, allowing cells to adapt to their natural morphology and potentially influencing signal transduction (E Frith et al., 2010). The PET/PDA/dECM carrier fabricated in this study exhibits an elevated proliferation rate following cell seeding, significantly higher than other reported three-dimensional scaffolds (such as polyester fiber porous scaffolds and alginate hydrogels) (Vaquero et al., 2017), which is beneficial for the long-term *in vitro* culture of MSCs. Furthermore, during cell cultivation in conjunction with the prepared carrier, it was observed that cell adhesion and growth occurred on both the anterior and posterior aspects of the substrate. Combined with the results of cell cytoskeleton staining, it is indicative that the presence of the ECM film on the PET/PDA/dECM carrier further facilitated cell adhesion and growth, with well-extended cells and a higher cell density. Cell growth on the carrier occurred from the surface gradually spreading inward, possibly driven by the active sites of PDA and dECM, with the carrier’s three-dimensional porous structure providing ample space for cell growth, conducive to long-term *in vitro* cell culture. The fibrous structure of the PET carrier used in this study partially simulated the cellular growth environment in the body, and the hydrophilic groups in PDA provided adhesion sites for cells. The presence of ECM proteins such as collagen and glycosaminoglycans enhanced the interaction between cells and the environment, promoting cell proliferation and secretory behavior (Rajasekaran et al., 2020).

The results of immunophenotypic analysis indicate that during the 14-day *in vitro* culture, the PET/PDA/dECM carrier provided excellent support for the growth of HUCMSCs, and the immunophenotype remained normal without undergoing differentiation. On the seventh day, there was an aberration in the cellular CD105 expression level within the PET/PDA group. This could be attributed to two main reasons: Firstly, cell aging (Tchkonia et al., 2013; Yin et al., 2019) might have occurred due to uneven initial cell seeding density on the three-dimensional carrier, leading to some cells being in prolonged isolation and prematurely aging. Additionally, other studies have shown that the downregulation of CD105 expression in UCMSCs might be related to multilineage differentiation, and its expression level correlates negatively with the degree of cell differentiation (Jin et al., 2009). Research by Wang et al. (2019) and Rim et al. (2012) has demonstrated that polydopamine coatings on material surfaces can promote MSCs’ osteogenic differentiation. Therefore, it can be inferred that polydopamine induced differentiation of HUCMSCs on PET fibers, resulting in the downregulation of CD105. However, in the PET/PDA/dECM carrier, the extracellular matrix coating covered the original polydopamine, partially attenuating the induction of differentiation by PDA. Moreover, some native biomolecules present in the dECM coating may also contribute to maintaining the stemness of MSCs, ensuring normal expression of surface markers (Rakian et al., 2015). On the 14th day, the downregulation of CD105 in the TCPS group might be due to prolonged contact inhibition leading to cell aging. The low-level expression of CD34 and HLA-DR in the PET/PDA group could be attributed to the accumulation of reactive oxygen species during cell culture, resulting in oxidative stress and stimulation of MSCs by pro-inflammatory cytokines (Dominici et al., 2006; Pourgholaminejad et al., 2016). However, other studies have indicated that the upregulation of HLA-DR expression does not affect the multipotent differentiation potential and immunomodulatory properties of MSCs (Grau-Vorster et al., 2019). Additionally, the further downregulation of CD105 expression in the PET/PDA group compared to day 7 suggests an increased degree of differentiation in HUCMSCs.

By conducting gene expression profiling of relevant factors in cells cultured on the three substrates, the results suggest that compared to the two-dimensional TCPS and PET/PDA carriers, the PET/PDA/dECM carrier significantly promotes the gene expression of HUCMSCs cytokines, especially HGF, Ang-1, KGF, and IL-10. The significant upregulation of IL-10 could be attributed to the accumulation of intracellular ROS on the carrier, promoting the secretion of the anti-inflammatory factor IL-10. In the PET/PDA carrier, the fibers’ surface contains numerous PDA particles, and Deng et al. (2021) found that polydopamine can significantly reduce intracellular reactive oxygen species due to the oxidative action of the reducing functional groups in PDA. The coverage of PDA on PET/PDA/dECM carrier by the decellularized extracellular matrix film led to a substantial expression of IL-10 by the cells on the PET/PDA/dECM carrier. Additionally, the upregulation of VEGF expression in the PET/PDA group indicates that the fiber structure in the carrier may promote the angiogenesis-related functional expression of HUCMSCs. The above results indicate that dECM can effectively promote the gene expression of relevant cytokines, which is consistent with similar findings in other studies (Huang et al., 2017; Talovic et al., 2019; Al Belushi et al., 2020). This is mainly attributed to the ECM’s composition, which effectively mimics the *in vivo* niche of cells, thereby facilitating cell-material interactions, and strengthening cellular metabolism and signal pathway expression (Guilak et al., 2009; Mruthyunjaya et al., 2010; Ode et al., 2010; He et al., 2013; Vidane et al., 2013; Wu et al., 2015).

As for the protein expression of relevant factors in cells cultured on the three substrates, a robust pro-secretory effect was similarly evident. However, at the 7th and 14th days, the protein expression levels within the TCPS group were significantly lower than the other two groups. This may be due to contact inhibition occurring in the TCPS group, while cells cultured on the three-dimensional (3D) carriers continue to exhibit rapid proliferation and increased protein expression. Follin et al. (2016) summarized a large number of studies and found that compared to two-dimensional cultures, three-dimensional cultures can enhance the paracrine immunomodulatory potential of MSCs, which is consistent with the increased cytokine protein expression in the PET carrier group. Additionally, in the PET/PDA group, the mRNA expression of certain factors (HGF, VEGF, KGF) was lower than in the TCPS group, while their protein expression was higher than in the TCPS group, indicating that mRNA expression levels in cells do not always exhibit a positive correlation with protein levels (Chen et al., 2002; Guo et al., 2008). Furthermore, it is noteworthy that the protein expression trends of certain cytokines, such as Ang-1, in HUCMSCs cultured on the composite carriers are not entirely consistent with their mRNA expression levels. This inconsistency can be attributed to the temporal and spatial asynchrony of gene transcription and translation, the inherent instability of mRNA which may degrade during extraction and storage, and the intricate regulation of mRNA translation into proteins, which also involves post-translational processing and modifications. As a result, discrepancies between mRNA and protein expression profiles can arise (Chen et al., 2002).

The ELISA results were in good agreement with qPCR and Western blot results, further demonstrating that PET/PDA/dECM can significantly promote the expression and secretion of active factors in HUCMSCs. Similar findings have been reported in other studies, such as Ciuffreda et al. (2018), who found that adding heparin to hydrogel scaffolds significantly enhanced the paracrine secretion of VEGF and bFGF by rBMSCs. Lin et al. (2015) also observed that the extracellular matrix scaffold derived from small intestine submucosa (SIS) significantly increased the secretion of vascular growth factors (HGF, VEGF, IGFBP, IL-8, etc.) by hMSCs.

The above results indicate that compared to the conventional two-dimensional culture system TCPS, both PET/PDA and PET/PDA/dECM carriers can effectively promote MSC proliferation and the gene and protein expression of key cytokines. Particularly, the PET/PDA/dECM carrier, with a significant amount of dECM components, showed superior expression of key cytokines compared to other experimental and control groups, fully demonstrating the advantages of this composite system in terms of biocompatibility.

The PET/PDA/dECM composite carrier system offers a higher specific surface area, a microenvironment closer to the *in vivo* conditions, and improved cell-to-cell interactions. These outstanding attributes make it a promising candidate for long-term three-dimensional *in vitro* cultivation of MSCs. Moreover, it holds great potential for applications in tissue engineering, drug screening, drug efficacy assessment, organ models, and more. Additionally, this system can be integrated with bioreactors to facilitate large-scale cell culture. Quality control and standardization of cell cultivation can be achieved through automated systems, aiding in cell expansion and the production of various products, including biopharmaceuticals, cell therapy products, and cell vaccines. Conclusive research findings underscore the successful integration of microcarriers with a bioreactor, achieving large-scale cell cultivation. Following a two-week cultivation period in the bioreactor, the cell population reached 3 × 10^8, demonstrating a commendable efficacy in *ex vivo* expansion (Li et al., 2020). However, it is important to note that the volume of microcarriers can pose limitations for large-scale cell culture. Developing a scaleable microcarrier production process, optimizing the production pipeline to increase microcarrier yield, and refining large-scale cultivation systems to ensure uniform distribution and stability of microcarriers are essential. Furthermore, streamlining cell culture processes and implementing appropriate quality assessment and standardization systems are vital steps to maximize yield and product quality. These efforts aim to ensure the performance and feasibility of microcarriers in large-scale cultivation applications.

## 5 Conclusion

In this study, PET was used as the substrate material, and PDA was grafted onto its surface, followed by coating with decellularized extracellular matrix (dECM) derived from lung tissue, resulting in the preparation of a three-dimensional composite scaffold called PET/PDA/dECM. Through characterization, the fabricated three-dimensional composite scaffold exhibited complete hydrophilicity, high porosity, and favorable mechanical properties, and it was rich in various ECM proteins, demonstrating excellent biocompatibility. Cell experiments indicated that compared to traditional two-dimensional culture, the prepared PET/PDA/dECM scaffold significantly promoted the adhesion and proliferation of HUCMSCs while maintaining their stemness and enhancing the expression of HUCMSCs’ cytokines at both the gene and protein levels.

In conclusion, the PET/PDA/dECM scaffold demonstrated excellent cell compatibility and holds promise for long-term *in vitro* culture of MSCs while preserving their stem cell phenotype. It could potentially be used for the large-scale production of MSCs and their paracrine products for clinical research and therapy.

## Data Availability

The original contributions presented in the study are included in the article/supplementary material, further inquiries can be directed to the corresponding author.
